# Interleukin 1α-Deficient Mice Have an Altered Gut Microbiota Leading to Protection from Dextran Sodium Sulfate-Induced Colitis

**DOI:** 10.1128/mSystems.00213-17

**Published:** 2018-05-08

**Authors:** Moran Nunberg, Nir Werbner, Hadar Neuman, Marina Bersudsky, Alex Braiman, Moshe Ben-Shoshan, Meirav Ben Izhak, Yoram Louzoun, Ron N. Apte, Elena Voronov, Omry Koren

**Affiliations:** a Shraga Segal Department of Microbiology and Immunology, Faculty of Health Sciences, Ben-Gurion University of the Negev, Beer Sheva, Israel; b The Cancer Research Center, Faculty of Health Sciences, Ben-Gurion University of the Negev, Beer Sheva, Israel; c Azrieli Faculty of Medicine, Bar Ilan University, Safed, Israel; d Faculty of Life Sciences, Bar-Ilan University, Ramat Gan, Israel; e Department of Mathematics, Bar-Ilan University, Ramat Gan, Israel; University of California, Irvine

**Keywords:** bacteria, IL-1α, microbiome, gut, inflammatory bowel disease

## Abstract

Inflammatory bowel diseases (IBD) are a group of chronic inflammatory disorders of the intestine, with as-yet-unclear etiologies, affecting over a million people in the United States alone. With the emergence of microbiome research, numerous studies have shown a connection between shifts in the gut microbiota composition (dysbiosis) and patterns of IBD development. In a previous study, we showed that interleukin 1α (IL-1α) deficiency in IL-1α knockout (KO) mice results in moderate dextran sodium sulfate (DSS)-induced colitis compared to that of wild-type (WT) mice, characterized by reduced inflammation and complete healing, as shown by parameters of weight loss, disease activity index (DAI) score, histology, and cytokine expression. In this study, we tested whether the protective effects of IL-1α deficiency on DSS-induced colitis correlate with changes in the gut microbiota and whether manipulation of the microbiota by cohousing can alter patterns of colon inflammation. We analyzed the gut microbiota composition in both control (WT) and IL-1α KO mice under steady-state homeostasis, during acute DSS-induced colitis, and after recovery using 16S rRNA next-generation sequencing. Additionally, we performed cohousing of both mouse groups and tested the effects on the microbiota and clinical outcomes. We demonstrate that host-derived IL-1α has a clear influence on gut microbiota composition, as well as on severity of DSS-induced acute colon inflammation. Cohousing both successfully changed the gut microbiota composition and increased the disease severity of IL-1α-deficient mice to levels similar to those of WT mice. This study shows a strong and novel correlation between IL-1α expression, microbiota composition, and clinical outcomes of DSS-induced colitis.

**IMPORTANCE** Here, we show a connection between IL-1α expression, microbiota composition, and clinical outcomes of DSS-induced colitis. Specifically, we show that the mild colitis symptoms seen in IL-1α-deficient mice following administration of DSS are correlated with the unique gut microbiota compositions of the mice. However, when these mice are exposed to WT microbiota by cohousing, their gut microbiota composition returns to resemble that of WT mice, and their disease severity increases significantly. As inflammatory bowel diseases are such common diseases, with limited effective treatments to date, there is a great need to better understand the interactions between microbiota composition, the immune system, and colitis. This study shows correlation between microbiota composition and DSS resistance; it may potentially lead to the development of improved probiotics for IBD treatment.

## Abstract (precis)

Here, we show a connection between IL-1α expression, microbiota composition, and clinical outcomes of DSS-induced colitis. Specifically, we show that the mild colitis symptoms seen in IL-1α-deficient mice following administration of DSS are correlated with the unique gut microbiota compositions of the mice. However, when these mice are exposed to WT microbiota by cohousing, their gut microbiota composition returns to resemble that of WT mice, and their disease severity increases significantly. As inflammatory bowel diseases are such common diseases, with limited effective treatments to date, there is a great need to better understand the interactions between microbiota composition, the immune system, and colitis. This study shows correlation between microbiota composition and DSS resistance; it may potentially lead to the development of improved probiotics for IBD treatment.

## INTRODUCTION

Inflammatory bowel diseases (IBD) are a group of chronic inflammatory disorders with yet-unclear etiologies that affect the small and large intestines (1). Crohn’s disease (CD) and ulcerative colitis (UC) are the two primary idiopathic forms of IBD in humans and feature mainly loss of epithelial and mucosal layer integrity, infiltration of immune cells, severe inflammation, bloody diarrhea, and an increased risk of developing colorectal cancer (2). Various models have been developed to induce colitis in mice that display clinical characteristics that are similar to those of humans with IBD (3). One of the most common models includes administration of dextran sodium sulfate (DSS) for several days, which induces acute colitis characterized by bloody diarrhea, crypt damage, and infiltration of inflammatory cells, similar to CD (4).

With the emergence of microbiome research in recent years, dysbiosis has been associated with development of IBD (5). Organisms including Escherichia coli, *Bacteroides*, *Enterococcus*, and *Klebsiella*, among various other commensal organisms, have been implicated in the pathogenesis of experimental and human IBD (5). Mechanistically, dysbiosis may lead to a proinflammatory state, further resulting in chronic inflammation in a susceptible host (6).

In previous studies, we showed that IL-1α deficiency moderates DSS-induced colitis with minimal inflammation and complete healing (7). IL-1α has a central role in the regulation of colon inflammation (7). Administration of DSS results in damage of intestinal epithelial cells (IECs) and leads to their necrosis and spillage of their cellular contents, including cell-associated IL-1α, into the extracellular microenvironment. This initiates the inflammatory response, with IL-1α acting as an alarmin, subsequently recruiting bone marrow-derived myeloid cells to the intestine and gut-associated lymphoid tissue (GALT), with activation of a potent local proinflammatory cytokine cascade. Monocytes/macrophages, as already indicated above, later infiltrate the damaged colon and act on microbial clearance, induce repair, and return to homeostasis by secreting IL-1β (7). Accordingly, patterns of colitis were exacerbated in IL-1β KO mice compared to those of WT mice. This is also one of the few reports showing *in vivo* differences between the two major IL-1 agonistic molecules, IL-1β and IL-1α, which bind to the same IL-1 signaling receptor. In a subsequent study, the role of IL-1α as a driver of DSS-induced colitis in IL-33-deficient mice has also been established (8).

In this study, we investigated whether the protective effects of IL-1α deficiency on DSS-induced colitis correlate with changes in the gut microbiota and whether manipulation of bacterial populations by cohousing can alter disease progression.

## RESULTS

### IL-1α KO mice harbor a unique intestinal microbiota.

**FIG 1:**
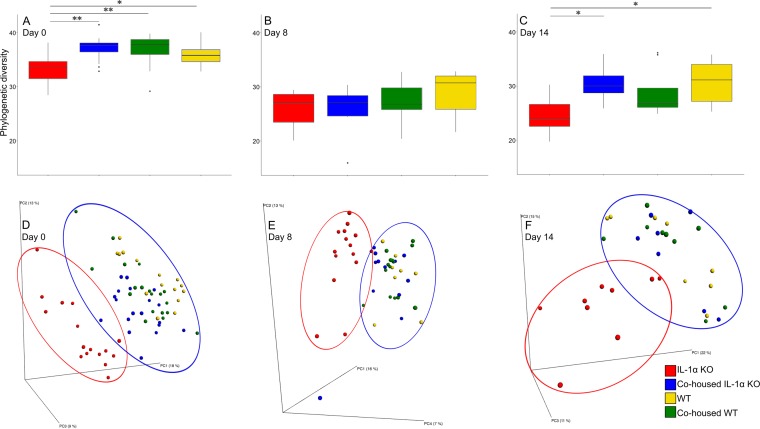
Alpha diversities and beta diversities differ between mouse groups. (A to C) Alpha diversities (Faith’s phylogenic diversity [PD]) before DSS treatment (day 0) (A), after 7 days of DSS (day 8) (B), and after 7 days of recovery (day 14) (C); (D to F) PCoA results of unweighted UniFrac distances on days 0, 8, and 14 for IL-1α KO, WT, cohoused IL-1α KO, and cohoused WT mice. *, *P* < 0.05; **, *P* < 0.01. PC2, principal component 2.

**FIG 2:**
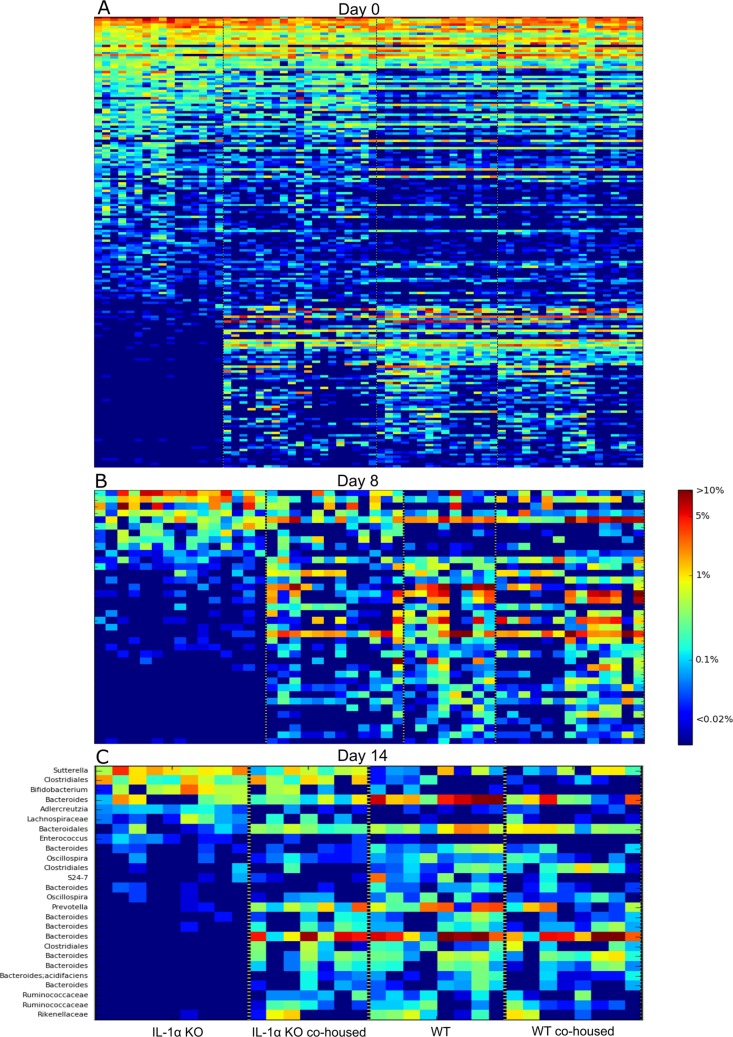
Heatmaps of differential OTUs. Heatmaps were generated using Heatsequer, identifying OTUs that differ between IL-1α and WT mice by abundance rank, occurrence, mean abundance, or frequency when present. Abundances of these differential OTUs in all groups are presented before DSS treatment (day 0) (A), after 7 days of DSS (day 8) (B), and after 7 days of recovery (day 14) (C). See Tables S1 and S2 in the supplemental material for taxonomic ranks for panels A and B.

To further understand the role of the microbiota in IBD, we first assessed whether IL-1α deficiency affects the gut microbiota. Indeed, clear differences were observed in steady-state homeostasis between IL-1α KO and WT mice in all parameters of microbial composition, including within-sample diversity (alpha diversity), between-sample diversity (beta diversity), and species abundance. In terms of alpha diversity, the microbiota of IL-1α KO mice was significantly less diverse than that of WT mice (*P* < 0.05) (Fig. 1A). As to beta diversity, UniFrac analysis demonstrated two separate clusters reflecting distinct differences in microbial compositions between WT and IL-1α KO mice (Fig. 1D). In analysis of taxonomical differences, there were many significant alterations in the abundances of families, genera, and even specific operational taxonomic units (OTUs) between IL-1α KO and WT mice, as shown in the heatmaps presented in Fig. 2A. More specifically, we found 9 families, 13 genera, and 190 OTUs to be significantly altered in IL-1α KO mice compared to WT mice (Fig. 2A; for a detailed taxonomical list, see Table S1 in the supplemental material). Among these, the relative abundances of members of the families *Coriobacteriaceae* and RF32 and of genera such as *Bacteroides*, AF12, and *Akkermansia* were considerably lower in IL-1α KO mice than in WT mice. On the other hand, the abundances of *Turicibacter*, *Ruminococcus*, and members of the family *Desulfovibrionaceae* were considerably higher in IL-1α KO mice than in WT mice. Taken together, these results reflect a dramatic effect of IL-1α on the gut microbiota, leading to significant alterations in its composition from that of WT mice.

10.1128/mSystems.00213-17.3 TABLE S1  Taxonomical ranks for Fig. 2A. Download TABLE S1, PDF file, 0.04 MB. Copyright © 2018 Nunberg et al. 2018 Nunberg et al. This content is distributed under the terms of the Creative Commons Attribution 4.0 International license.

10.1128/mSystems.00213-17.4 TABLE S2  Taxonomical ranks for Fig. 2B. Download TABLE S2, PDF file, 0.02 MB. Copyright © 2018 Nunberg et al. 2018 Nunberg et al. This content is distributed under the terms of the Creative Commons Attribution 4.0 International license.

### Cohousing of WT and IL-1α KO mice alters their microbial profiles.

We wished to test whether the differences seen in microbial composition between WT and IL-1α KO mice can be modulated by cohousing of the different mice. Since mice are coprophagic, cohousing exposes them to each other’s microbiota, and it is thus expected that their gut microbial compositions will become more similar and disease susceptibility will be altered, as was recently demonstrated by Surana and Kasper (9). Therefore, WT and IL-1α KO mice were cohoused for 30 days (termed cohoused WT and cohoused IL-1α KO mice), and their microbiotas were assessed thereafter.

We observed that after 30 days of cohousing, there were clear differences between the evaluated groups, as seen by principal-coordinate analysis (PCoA) clustering (Fig. 1D). More specifically, there appeared to be a gradient of microbial compositions between the IL-1α KO and WT mice, with the microbial compositions of cohoused groups located in between the two extremes. The cohoused IL-1α KO mice showed a shift toward the WT control mice and in fact were closer to the WT controls than to the control IL-1α KO mice. Similarly, the cohoused WT mice shifted toward the IL-1α KO mice but remained closer to the WT control group. In order to further validate the differences among the samples, the multivariate analysis of variance (MANOVA) test was performed on the two-dimensional projections of all samples on the first two principal-component analysis (PCA) vectors. The test was significant at the *P* level of <0.0001. A *post hoc* Hotelling T2 test was then performed to compare the IL-1α KO to the WT mice. Only noncohoused mice showed a significant difference in microbial composition between the IL-1α KO and the WT mice at day 0 (*P* was 0.0068 for the noncohoused mice, and differences were not significant for the cohoused mice) (Table S3). A linear discriminant analysis (LDA) was then performed over the PCA projection of the OTUs during the first day, and the noncohoused IL-1α KO mice were completely distinct from the WT mice, while the cohoused animals overlapped the WT animals. Similar results were obtained for all other days (Fig. S1).

10.1128/mSystems.00213-17.1 FIG S1  A linear discriminant analysis (LDA) was performed to detect the axis separating WT from IL-1α mice. The bars represent a histogram of the projection of the microbiome profile of each mouse on this axis, where positive values represent the WT mice direction (yellow bars) and negative values the IL-1α mice direction (blue bars). Download FIG S1, PDF file, 1.4 MB. Copyright © 2018 Nunberg et al. 2018 Nunberg et al. This content is distributed under the terms of the Creative Commons Attribution 4.0 International license.

10.1128/mSystems.00213-17.4 TABLE S3  *P* values of the effect of cohousing on microbiome expression patterns. Download TABLE S3, PDF file, 0.01 MB. Copyright © 2018 Nunberg et al. 2018 Nunberg et al. This content is distributed under the terms of the Creative Commons Attribution 4.0 International license.

In terms of taxonomic abundance, both cohoused groups presented abundance patterns resembling those of the WT and different from those of the IL-1α KO mice, as seen in Fig. 2A. These results reflect a dramatic effect of the IL-1α deficiency on the gut microbial composition, leading to significant alterations from that of WT mice. This shift in the gut microbiota of the cohoused mice was also observed in measurements of alpha diversity (Fig. 1A).

### Cohousing of IL-1α KO with WT mice diminishes their resistance to DSS-induced colitis.

**FIG 3:**
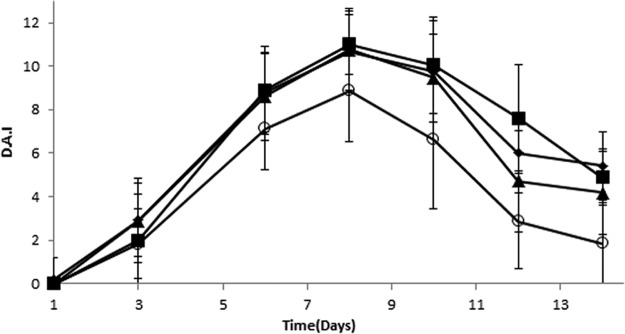
Clinical responses of DSS-treated cohoused mice. The DAI was scored daily for WT mice, IL‑1α KO mice, cohoused IL-1α, and cohoused WT mice after DSS treatment (*n =* 8 for all groups). Data are means ± SEM from four independent experiments. *, *P* < 0.05; **, *P* < 0.001 (*t* test) between IL-1α KO mice and the other groups.

As we have previously shown, IL-1α KO mice are resistant to DSS-induced colitis (7). After demonstrating their unique microbial profiles, which are altered by cohousing, we wished to test whether changes in microbiota can affect their response to DSS-induced colitis and epithelial barrier (EB) repair. We were especially interested whether the observed alterations in the microbiota in the cohoused groups would affect their resistance to DSS-induced colitis, compared to that of their noncohoused counterparts. To this end, naive WT and IL-1α KO mice, as well as mice from both cohoused groups, were exposed to DSS administration for 7 days. The disease activity index (DAI) score and survival rate were determined during DSS treatment and during 7 additional days of recovery. In accordance with our previous results, WT mice showed a severe pattern of colon inflammation, resulting in a high DAI (day 8) (Fig. 3). In contrast, the IL-1α KO mice showed high resistance to the DSS treatment, with 95% survival throughout the experiment, and significantly lower DAI scores than those of the WT mice starting from day 6. The administration of DSS to both cohoused WT and cohoused IL-1α KO mice led to patterns of disease similar to those observed in WT mice (Fig. 3).

**FIG 4:**
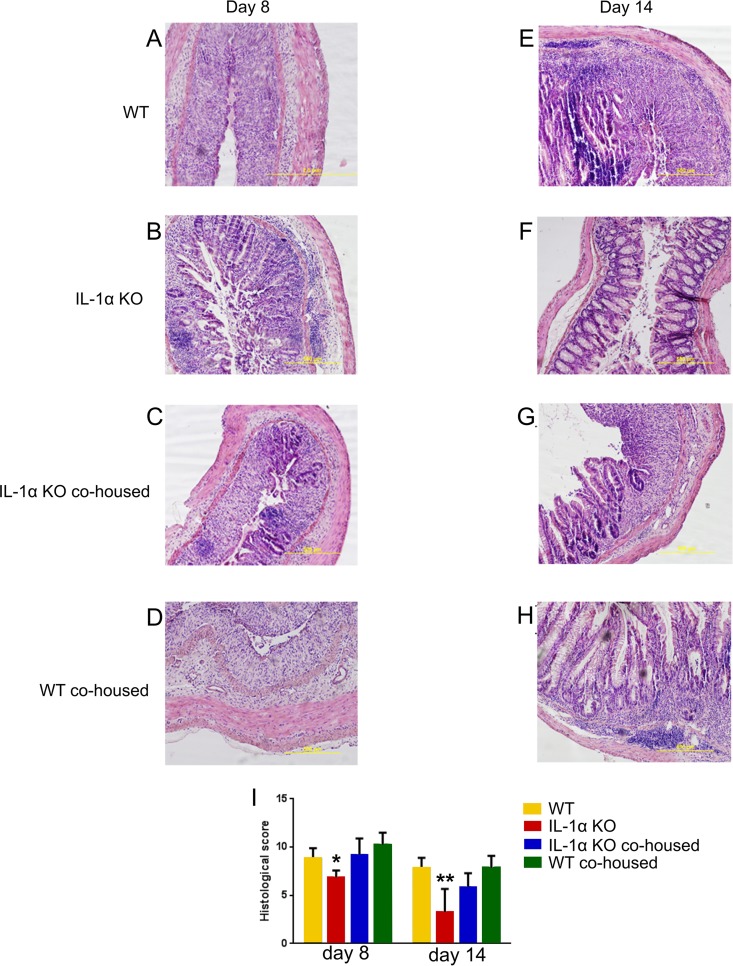
Histological changes in mouse colons after DSS treatment. Representative photomicrographs of colons of DSS-treated WT, IL-1α KO, and cohoused mice, sacrificed on day 8 or 14 (H&E staining, ×200 magnification). (Left) Day 8; (right) day 14. (A and E) WT mice; (B and F) IL-1α KO mice; (C and G) IL-1α cohoused mice; (D and H) cohoused WT mice; (I) means ± SEM of histological scores of colons on days 8 or 14 after the initiation of DSS treatment (8 mice/group). The data are from five independent experiments. *, *P* < 0.05; **, *P* < 0.001 (*t* test).

In order to evaluate the severity of the colon damage, distal colon sections stained with hematoxylin and eosin (H&E) were evaluated. No differences in colon structure were detected between WT and IL-1α KO mice on day 0 after cohousing, without DSS administration (Fig. S2). Nevertheless, as expected from our previous findings, a more severe colon inflammation was observed in DSS-treated WT mice than in IL-1α KO mice, as evident by histological scores. Consistently with the survival data, both cohoused groups revealed an intermediate state of colon inflammation compared to that of the noncohoused groups (Fig. 4A to D).

10.1128/mSystems.00213-17.2 FIG S2  Representative photomicrographs of H&E tissue sections from colons on day 0. Download FIG S2, PDF file, 0.02 MB. Copyright © 2018 Nunberg et al. 2018 Nunberg et al. This content is distributed under the terms of the Creative Commons Attribution 4.0 International license.

On day 14, almost complete recovery was observed in IL-1α KO mice, but not in WT mice. Cohoused mice showed an intermediate state of histological changes, with better regeneration and less inflammation than those of DSS-treated WT mice (Fig. 4E to H).

Overall, it appears that the deficiency of IL-1α leads to a more moderate form of colitis; however, exposure to WT microbiota exacerbates disease in this group of mice (Fig. 4I).

**FIG 5:**
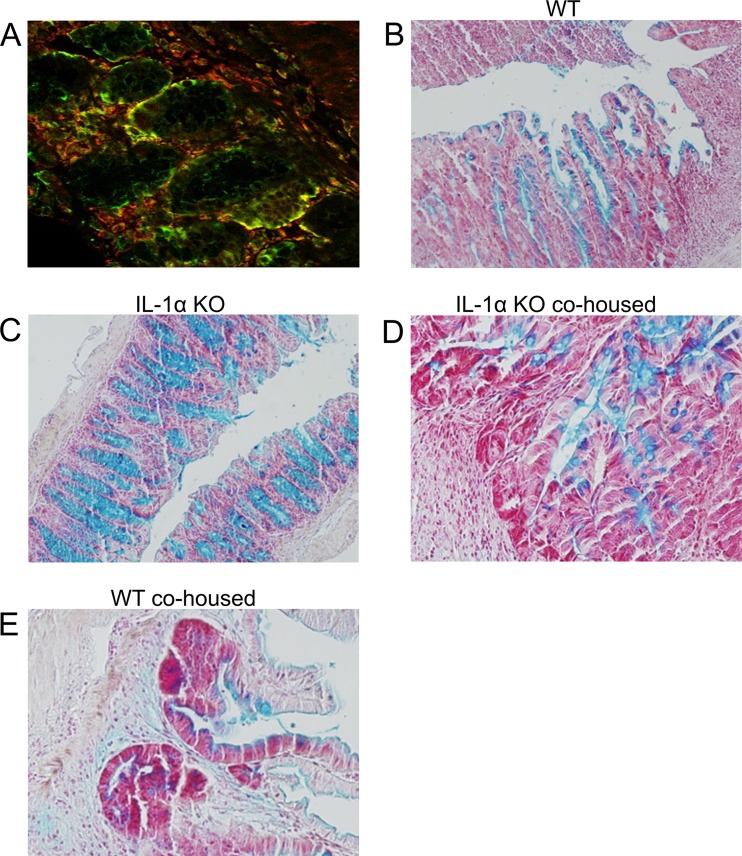
Expression of IL-1α in goblet cells and their destruction during DSS-induced colitis. (A) Colon tissue from control WT mice costained with anti-IL-1α (green) and antimucin (red) antibodies (×60 magnification). Photomicrographs of colon sections from WT and IL-1α KO mice and cohoused mice were obtained on day 14 after initiation of DSS treatment and were stained with alcian blue to identify goblet cells. (B) WT mice; (C) IL-1α KO mice; (D) cohoused IL-1α mice; (E) cohoused WT mice.

To further assess the mechanisms by which the microbiota may affect the DSS response in IL-1α KO mice, we first evaluated IL-1α in different colon epithelial cell types. We have previously shown that IL-1α is located intracellularly in colon crypts and released outside by epithelial cells during necrosis (7). In this study, we stained IL-1α in resident colon cells and found its strong colocalization with the mucin-producing goblet cells (Fig. 5A). These findings suggest that goblet cells are the main producers of IL-1α in the colon. Next, we stained goblet cells in WT mice and mice deficient in IL-1α under homeostatic conditions and after DSS administration. No differences were observed in the abundances of goblet cells between untreated WT and IL-1α KO mice (data not shown). Nevertheless, even after termination of DSS treatment, a significant decrease in goblet cells was detected in WT mice, as well as in both cohoused groups of mice. In contrast, goblet cells were not significantly affected after recovery from colitis in IL-1α-deficient mice (Fig. 5B to E). This finding demonstrates that the IL-1α-dependent sensitivity to DSS in WT mice probably involves damage of goblet cells.

**FIG 6:**
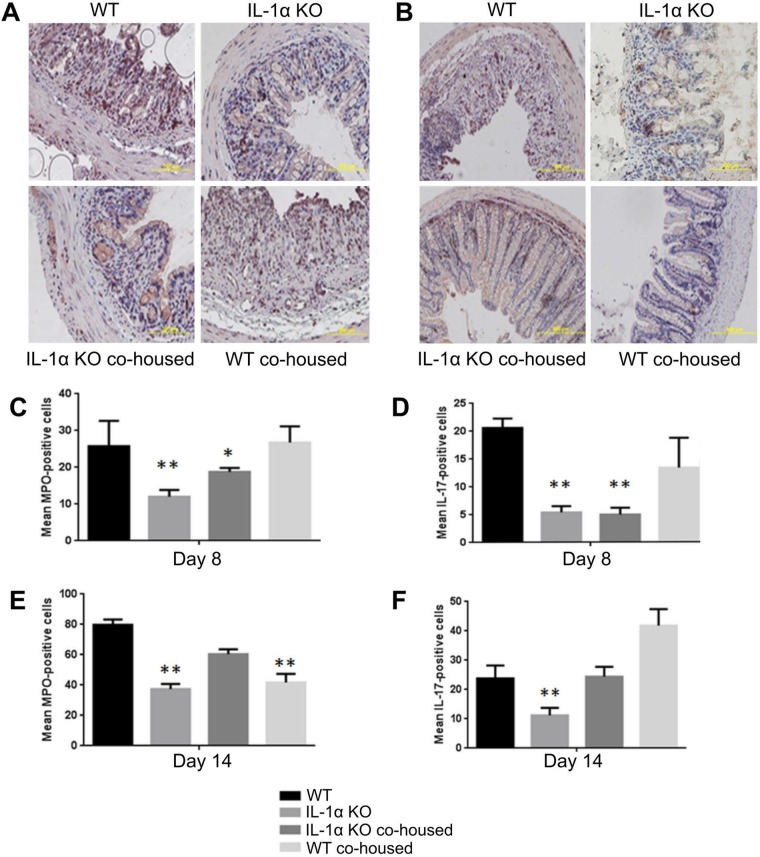
Effects of IL-1α on the composition of infiltrating proinflammatory cells in inflamed colons during DSS-induced colitis. (A and B) Colon tissues from DSS-treated mice were obtained on days 8 and 14 and were stained with anti-MPO and anti-IL-17 for IHC analyses. The numbers of MPO-positive cells (C and E) and IL-17-positive cells (D and F) were counted in 6 random fields under a microscope, and the means of the numbers of positive cells are shown. Results presented are the averages of results from three independent experiments (*, *P* < 0.05;* t* test).

We next assessed the immune cells infiltrating the colon in DSS-treated mice. We analyzed the presence of neutrophils, as they are the most abundant leukocytes in an inflamed colon, using immunohistochemistry (IHC) staining with antimyeloperoxidase (anti-MPO) antibodies. After DSS administration, on day 8, an abundant recruitment of MPO-positive cells was observed in WT mice and cohoused WT mice, but significantly lower levels were observed in IL-1α KO and cohoused IL-1α KO mice (Fig. 6A and C). After recovery (day 14), in all groups of mice, the number of MPO-positive cells increased, but as on day 8, IL-1α KO mice presented significantly lower levels of MPO-positive cells than those of WT mice (Fig. 6E). Interestingly, the patterns of cohoused mice more closely resembled those of IL-1α KO mice.

Analysis of IL-17-positive proinflammatory T cells revealed their significant recruitment in WT mice compared to that in IL-1α KO mice at both 8 and 14 days (Fig. 6B and D). Cohousing did not affect these levels at day 8; however, at day 14, the number of IL-17-positive cells was higher in both cohoused groups as well as in WT mice, though not in IL-1α KO mice (Fig. 6F).

### Microbial profiles of IL-1α KO mice differ from those of WT and cohoused mice throughout DSS treatment and recovery.

Finally, we wished to test whether the resistance to DSS treatment in IL-1α KO mice is correlated with a distinct microbiota; to this end, we compared the microbiotas of all mouse groups after DSS treatment and during recovery. While the alpha diversity did not differ among mouse groups on day 8 (Fig. 1B), it was lower than on day 0 in all groups. The IL-1α KO mice exhibited significantly less diversity than both WT and cohoused IL-1α KO groups at day 14 (Fig. 1C). The cohoused groups were indistinguishable from the WT mice in terms of beta diversity, while the IL-1α KO mice differed, reinforcing the notion that the WT microbiota dominated in the cohoused groups (Fig. 1E and F).

Additionally, following DSS treatment, significant alterations in microbial composition between WT and IL-1α KO mice remained evident, as seen by differences in the relative abundances of diverse genera, families, and orders (Fig. 2B). At the species level, the abundances of two species appeared to be significantly different; Ruminococcus gnavus showed higher abundance and Akkermansia muciniphila showed lower abundance in IL-1α KO mice than in WT mice (Fig. 2B). During recovery (day 14), there remained significant differences in microbial abundances between IL-1α KO and WT mice (Fig. 2C). At day 8, among the significantly different genera observed between groups were *Enterococcus* and *Bacteroides*, and among the families were *S24-7*, *Ruminococcaceae*, *Rikenellaceae*, *Coriobacteriaceae*, and *Lachnospiraceae*. Additionally, at this time point, several genera, including *Sutterella*, *Adlercreutzia*, *and Bifidobacterium*, appeared more abundant in the IL-1α KO mice than in the WT mice. Other genera, including *Bacteroides*, *Prevotella*, and *Oscillospira*, appeared less abundant in the IL-1α KO mice. At the family level, *S24-7*, *Ruminococcaceae*, *Rikenellaceae*, and *Coriobacteriaceae* were less abundant in IL-1α KO mice, whereas *Lachnospiraceae* were more abundant (Fig. 2C).

**FIG 7:**
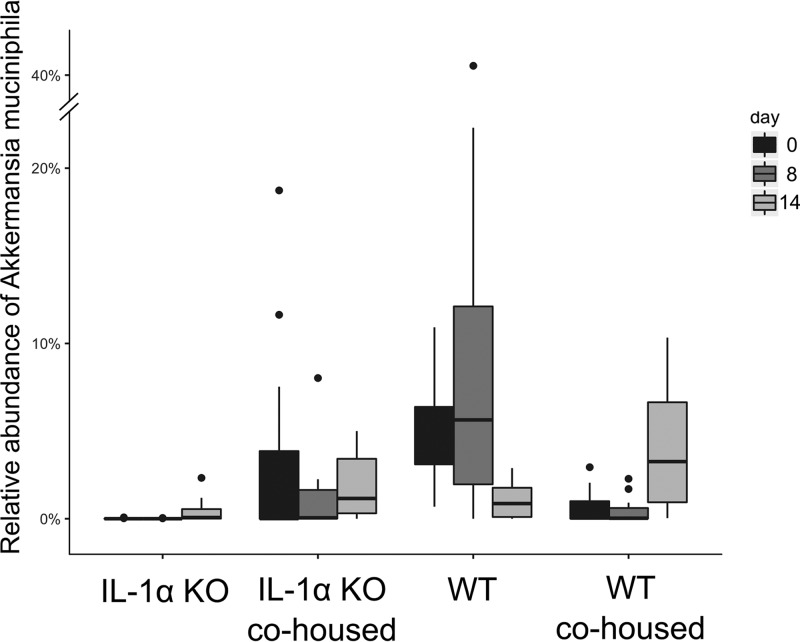
Relative abundances of Akkermansia muciniphila organisms in all mouse groups. The fraction of Akkermansia muciniphila organisms of the total number of bacteria was calculated for each mouse group at days 0, 8, and 14 and is presented in boxplots. Whiskers represent the full range, while outliers are presented as dots. Differences are significant on day 0 (*P* < 0.001) and day 8 (*P* < 0.05, Kruskal-Wallis test, FDR corrected).

The similarity of microbial abundance patterns between cohoused groups and WT mice, in contrast to IL-1α KO mice, was maintained following DSS treatment on day 8 (Fig. 2B) and recovery on day 14 (Fig. 2C). Most significantly, both cohoused groups had noticeable abundance levels of Akkermansia muciniphila throughout DSS administration, as did the nontreated WT mice but not the IL-1α KO mice, in which the relative abundance was very low (Fig. 7). These results prove that cohousing greatly alters the microbial communities of both genotypes, shifting the gut microbial profile of IL-1α KO mice to a profile more closely resembling that of the WT mice.

## DISCUSSION

We previously demonstrated that IL-1α acts as a proinflammatory modulator of DSS-induced colitis (7). Accordingly, separately raised WT mice manifested a severe form of DSS-induced colitis, whereas IL-1α KO mice displayed a more resistant phenotype. Similar disease patterns were observed in conditional KO mice lacking IL-1α only in colon epithelial cells, indicating that intestinal epithelial cell-derived IL-1α is the driver of induction of DSS-induced colitis (7). The role of IL-1α in the initiation and maintenance of colitis was also recently confirmed by Malik et al. (8). As clearly observed in histological studies, IL-1α KO mice exhibited less inflammation and better, if not complete, repair of crypt structures after termination of DSS treatment than WT mice, in which resolution of damage occurred later in the disease course.

Our goal in this study was to determine whether the protective effects of IL-1α deficiency on DSS-induced colitis correlate with changes in the gut microbiota and whether cohousing can modulate disease outcomes. We chose the cohousing method, due to its simplicity and effectiveness in manipulating the microbiota (10). Since mice practice coprophagy (11), fecal microbes are transferred from one mouse to the other by simply sharing housing, without the stress caused by a gavage procedure.

Initially, we tested whether there were noticeable differences between the fecal microbiotas of naive WT and IL-1α KO mice. It was striking to observe that even though both types of inbred strains of mice are reared under the same specific-pathogen-free (SPF) conditions in our animal facility, their microbiotas are *a priori* different. Indeed, we found lower alpha diversity in IL-1α KO mice, as well as significant differences in beta diversity and taxonomy between groups. However, after 30 days of cohousing, these differences were much less significant. The microbiotas of both IL-1α KO and cohoused WT mice resembled that of WT animals, as demonstrated by the significantly higher alpha diversity than that of IL-1α KO mice and clustering with the WT samples in the PCoA plot. DSS treatment lowered phylogenetic diversity in all groups, leading to the lack of significant differences between them on day 8 and to a lesser extent on day 14. The microbiotas of both cohoused groups remained clustered closer to that of the WT mice than to that of the IL-1α KO mice at both 8 and 14 days. Similarly, the relative abundances of several bacterial genera and families remained significantly different between IL-1α KO mice and all other groups.

We found a much lower abundance of A. muciniphila in IL-1α KO mice than in all other groups, both before and after exposure to DSS. A. muciniphila is an anaerobic, mucus-degrading bacterium (12) that was recently associated with severity of colitis (13). Thus, it was recently demonstrated that levels of A. muciniphila are increased in DSS-treated mice and exacerbate gut inflammation (14), perhaps by mucin degradation, thereby enabling access of luminal microbes or their derived antigens to the submucosa and possibly also to deeper layers. Most recently, in a model of IL-10-deficient mice, under housing conditions where no spontaneous colitis develops, administration of A. muciniphila to such SPF-reared mice or monocolonization of IL-10-deficient germfree mice with A. muciniphila resulted in colitis induction. This directly points to the dominant colitogenic activity of A. muciniphila (15). Moreover, high levels of A. muciniphila were reported in IL-33-deficient mice and correlated with elevated expression of IL-1α, which was essential for induction of a severe form of colitis (8). In this experimental system, in accordance with our results, IL-1α is an initiator of the disease. Indeed, IL-1α of intestinal epithelial cell origin serves as an alarm cytokine that initiates and propagates the inflammatory response by activation of the local cytokine cascade and promotion of cell infiltration into the affected site (16). This fits our current observations, in which in WT mice, high abundance of A. muciniphila correlated with disease severity, while in IL-1α KO mice, this bacterium was almost absent. Indeed, in IL-1α KO mice, we found less damage to goblet cells, one of the main producer cells of IL-1α, than in WT and cohoused mice. This suggests that IL-1α is actively involved in colon inflammation through its release from the destruction of epithelial cells, phenomena that are absent in IL-1α KO mice but present in WT and cohoused mice.

In correlation with the microbiota changes, cohousing also exacerbated other parameters of colitis in IL-1α KO mice as seen by DAI, survival, and histological scores. Cohousing did not induce any behavioral changes. Cohoused groups exhibited an intermediate state compared to those of both separately raised groups of mice. These results clearly show that an alteration in colitis outcome in the cohoused mice was possibly influenced by microbial changes.

Variations in the microbiota, including those induced by cohousing experiments, have previously been shown to modulate the process of DSS-induced colitis and colorectal cancer development in mice lacking different cytokines and other components of innate immunity (pattern recognition receptors, inflammasome components) (17–23). Thus, for example, IL-33-deficient mice with severe patterns of colitis showed improved disease parameters, including colon length, histology score, and weight loss, when cohoused with WT mice. Expression of IL-33 is usually protective for gut inflammation (8). These changes were also correlated with a decrease in the abundance of A. muciniphila (8).

The involvement of specific bacteria in determining the fate of colitis is usually not clear. The mode of complex interactions among the mix of bacteria and their products with immune/inflammatory cells in the colon determines the outcome of colitis. Whereas some studies show favorable effects of Akkermansia muciniphila on colon inflammation, others show that A. muciniphila exacerbates colon inflammation, degrading mucin, which facilitates the access of luminal antigens and bacteria into the colon internal layers (15, 24, 25). This discrepancy remains as of now unclear; it is hoped that further understanding of the complex host-microbiota interactions will resolve it.

During the recovery period, we observed in cohoused IL-1α KO and IL-1α KO mice an increase in several other species, such as *Bifidobacterium*, known to have anti-inflammatory properties, which may play a role in improving the DSS-induced response (26). *Prevotella* is another genus that has been shown to be pathogenic in the colitis model (19). Our results also demonstrate that *Prevotella* is significantly reduced in IL-1α KO mice but that it is abundant in WT and cohoused mice. Thus, our results demonstrate a strong correlation between *Prevotella* and A. muciniphila levels and disease severity.

Next, we wished to test whether cohousing alters the DSS-induced immune/inflammatory response in the colon. DSS disrupts intestinal epithelia, causing the release of preformed IL-1α from necrotic cells. As IL-1α serves as an alarmin, it induces the production of itself and other proinflammatory cytokines, mainly by infiltrating myeloid cells that are recruited into the colon, which further propagate the inflammatory response (27, 28). We have previously shown that the alarmin function of IL-1α initially induces recruitment of neutrophils but that IL-1β is secreted later by recruited macrophages and propagates the inflammatory response (28). Accordingly, the number of MPO-positive cells was significantly higher in WT noncohoused mice than in IL-1α KO mice. Interestingly, even though the patterns of disease of cohoused mice were similar to those in WT mice, infiltration of MPO-positive cells resembled those of IL-1α KO mice on days 8 and 14. In contrast, infiltration of other proinflammatory cells, such as Th17, which were decreased in IL-1α KO mice during DSS treatment compared to levels in WT mice, were increased in both cohoused groups, observed mainly during the recovery period. These results support recent findings demonstrating the effects of specific bacteria on adaptive immune homeostasis and disease (29). For example, segmented filamentous bacteria (SFB) promote the development of Th17 cells, while Helicobacter hepaticus and some *Clostiridia* species affect differentiation of naive T cells into T regulator cells (30–33). The relationship between the presence of specific bacteria and the lack of cytokines in KO mice is largely unexplained. The use of gnotobiotic mice will enable resolving this issue. IL-1α is expressed in epithelial cells in its cell-associated form and thus may affect patterns of microbiota colonization or induce dysbiosis by mechanisms not yet known but being studied in our laboratory.

### Conclusions.

We demonstrate here that IL-1α has a clear influence on the gut microbiota composition, as well as on the severity of colitis. Cohousing was shown to change both the gut microbiota and patterns of disease progression. These results highlight the fact that some of the DSS insensitivity of the IL-1α KO mice were nondirect and, rather, mediated by the composition of the microbiota. Cohousing did not cause IL-1α KO mice to respond to DSS in a manner similar to that of WT mice, indicating that additional pathways, besides the microbiota, are involved in the pathogenesis of colitis. Nonetheless, effects on microbial composition were evident in phylogenetic diversity, between-species diversity, and the relative abundances of specific bacterial species. Effects on disease severity were demonstrated by the DAI score, histological score, and alterations in neutrophil and Th17 cell responses. Cohousing revealed that exposure of IL-1α KO mice to WT microbiota had a strong effect, leading to loss of protection from DSS, an inflammatory response, and severe disease outcomes similar to those observed in WT mice. In contrast, the effects of exposure to bacteria from IL-1α KO mice did not greatly affect microbial composition and did not alter disease progression in WT animals. This suggests that the lack of certain bacteria, as opposed to the gain of others, may play a role in IL-1α KO resistance to DSS. Overall, we show in this study a strong and novel correlation between IL-1α function, microbial composition, and clinical outcomes of DSS-induced colitis. Nevertheless, more research is needed to prove causation and exact interactions between cell-associated IL-1α, microbes, and cellular/cytokine pathways in the pathogenesis of colitis. While we identify some potentially involved microbial genera and families, their roles in relation to the DSS inflammatory response must be validated further and are the subject of our current studies.

## MATERIALS AND METHODS

### Mice.

C57BL/6 WT mice were initially purchased from Harlan Laboratories (Rehovot, Israel). IL-1α KO mice were generated in the laboratory of Y. Iwakura (34). These mice were backcrossed to C57BL/6 mice for more than 8 generations and are homozygous for the relevant mutation. Mice were bred and maintained under the same conditions at the Animal Facilities of the Faculty of Health Sciences, Ben-Gurion University, Beer Sheva, Israel. Animal studies were approved by the Animal Care Committee of Ben-Gurion University.

For cohousing experiments, IL-1α KO mice were maintained together with WT mice for 1 month and then separated for further analysis.

### DSS-induced colitis model.

A solution of 2.5% DSS (36 to 50,000 kDa; MP Biomedicals, Solon, OH) was administered in the drinking water of 8-week-old female mice for 7 days, followed by regular water for an additional 7 days. Survival and clinical signs of colitis were recorded daily, and the disease activity index (DAI), which includes weight loss, stool consistency, and occult blood in the stool, was determined. Occult/gross bleeding was measured using the Cenogenics stool blood test (SB-21; Cenogenics Corporation, Morganville, NJ). Weight loss was calculated as the percentage change relative to the body weight before initiation of the experiment.

Fecal samples were collected before DSS administration (day 0), at the end of DSS treatment (day 8), and after a week of recovery (day 14) and frozen immediately for further microbiota analysis.

### DNA extraction, amplification, and sequencing.

DNA was extracted using the Powersoil DNA extraction kit (MoBio, Carlsbad, CA). The 16S rRNA gene was amplified by PCR using PrimeSTAR Max DNA polymerase (TaKaRa, Mountain View, CA). The PCR consisted of 30 cycles of denaturation (95°C), annealing (55°C), and extension (72°C), with final elongation at 72°C. Primers for the variable V4 region (515F to 806R) were used, with overhangs, including sample-specific barcodes and Illumina adapters, as described previously (35). Amplicons were cleaned using AMPURE XP magnetic beads (Beckman Coulter, Inc., Indianapolis, IN) according to the manufacturer’s protocol. DNA concentration was quantified using the Quant-iT PicoGreen double-stranded DNA assay (Thermo, Fisher, Waltham, MA). Equimolar amounts of DNA were pooled from each sample to ensure equal read depths. After being cleaned on a 2% agarose E-Gel (Thermo, Fisher, Waltham, MA), DNA was sequenced on the Illumina Miseq platform at the Faculty of Medicine Genomic Center (Bar Ilan University, Safed, Israel). Successful sequencing was achieved for 151 samples: 40 IL-1α KO mice (16, 15, and 9 mice for days 0, 8, and 14, respectively), 31 WT mice (15, 8, and 8 mice), 38 cohoused IL-1α KO mice (19, 12, and 7 mice), and 42 cohoused WT mice (19, 14, and 9 mice).

### Bacterial composition analysis and bioinformatic methods.

Sequencing reads were quality filtered and trimmed using Trimmomatic (36). Further analysis, including paired-end joining, demultiplexing, chimera checking (using USEARCH [37]), and open-reference OTU picking, was done using QIIME (version 1.8.0) (38). OTUs that were less than 0.01% of total reads were discarded. Heatmaps were generated using Heatsequer (https://github.com/amnona/heatsequer). Differential OTUs were identified using four methods: abundance rank sum, occurrence in samples, difference in means, and mean abundance only in samples in which the OTU was present. Statistical analysis was done using 999 permutations and a false-discovery rate (FDR) of 0.1.

### Histological studies.

Colons were excised on days 0, 8, or 14 and embedded in paraffin by following a standard protocol (7). The severity of colitis in H&E-stained sections was assessed in a blind manner by a pathologist as previously described by us (7). The score for each parameter was added to obtain a total histological injury score.

### Immunohistochemistry and immunofluorescence staining.

The following primary antibodies were used: rabbit polyclonal anti-MPO (Abcam, Inc., Cambridge, United Kingdom) and polyclonal rabbit-anti-mouse IL-17 (Santa Cruz Biotechnology, Inc.).

The Universal ImmPRESS kit (Vector Laboratories, Burlingame, CA) was used for secondary staining for immunohistochemistry, and visualization was performed using 3-amino-9-ethylcarbazole (AEC) as a substrate (Zymed Laboratories, San Francisco, CA). The numbers of IL-17- or MPO-positive cells were counted in stained sections in six randomly chosen fields (magnification, ×400), and the results are presented as averages. Goblet cells were stained with the commercial alcian blue kit (Bio-Optica, Milan, Italy).

For immunofluorescence studies, we used goat anti-mouse IL-1α (R&D, Minneapolis, MN, USA) and rabbit polyclonal anti-MUC2 (antimucin) antibodies (Abcam, Inc., Cambridge, England). Secondary antibodies were conjugated with Cy3 and Cy5 (Jackson ImmunoResearch, West Grove, PA). Sections were examined under a Zeiss laser-scanning confocal microscope.

### Statistical analysis.

Each experiment was performed 2 to 6 times, and each experimental group consisted of 6 to 12 mice. Data are expressed as means ± standard errors of the means (SEM). Differences were analyzed using Student’s *t* test and one-way ANOVA. Survival curves were calculated using the Kaplan-Meier method. A linear discriminant analysis (LDA) was performed on the first five dimensions of the PCA projection of the normalized OTUs, where each OTU was log transformed, with a minimal value of 0.01 added to all OTUs to avoid 0 values in the log. The Z scores of logged OTUs were then determined by removing the average and dividing by the standard deviation for each sample and for each OTU. The LDA was performed on the day 0 for WT mice versus day 0 for IL-1α KO. All values were then projected on the same LDA vector.

A MANOVA test was performed on the projection of the same values on the first two PCA vectors (to limit the dimensionality of the data), and a *post hoc* Hotelling test was performed on the same projections.

All *P* values were corrected through a Benjamini-Hochberg FDR.

### Ethical approval.

Mice were bred and maintained at the Animal Facilities of the Faculty of Health Sciences, Ben-Gurion University, Beer Sheva, Israel. Animal studies were approved by the Animal Care Committee of Ben-Gurion University.

### Data availability.

The 16S rRNA gene sequence data have been deposited in the BioProject database under accession number PRJEB20762. All data related to the study can be downloaded from QIITA under study ID 11123.
