# Supplementary material for: Interleukin 1α-Deficient Mice Have an Altered Gut Microbiota Leading to Protection from Dextran Sodium Sulfate-Induced Colitis
Source: mSystems. 2018 May 8;3(3):e00213-17. doi: 10.1128/mSystems.00213-17 (PMC5940968; doi:10.1128/mSystems.00213-17)
Supplement: TABLE S1 [file sys003182227st1.pdf]

	Phylum	class	order	family	genus	species	Lowest taxonomy identified
1	Firmicutes	Clostridia	Clostridiales	Ruminococcaceae	Oscillospira		Oscillospira
2	Firmicutes	Clostridia	Clostridiales	Ruminococcaceae	Ruminococcus		Ruminococcus
3	Bacteroidetes	Bacteroidia	Bacteroidales	Bacteroidaceae	Bacteroides	ovatus	Bacteroides ovatus
4	Bacteroidetes	Bacteroidia	Bacteroidales	Bacteroidaceae	Bacteroides		Bacteroides
5	Bacteroidetes	Bacteroidia	Bacteroidales	Bacteroidaceae	Bacteroides	ovatus	Bacteroides ovatus
6	Bacteroidetes	Bacteroidia	Bacteroidales	Bacteroidaceae	Bacteroides		Bacteroides
7	Firmicutes	Clostridia	Clostridiales	Lachnospiraceae	Ruminococcus	gnavus	Ruminococcus gnavus
8	Firmicutes	Clostridia	Clostridiales				Clostridiales
9	Firmicutes	Clostridia	Clostridiales				Clostridiales
10	Firmicutes	Clostridia	Clostridiales				Clostridiales
11	Firmicutes	Clostridia	Clostridiales				Clostridiales
12	Firmicutes	Clostridia	Clostridiales	Lachnospiraceae	Dorea		Dorea
13	Firmicutes	Clostridia	Clostridiales				Clostridiales
14	Cyanobacteria	4C0d-2	YS2				YS2
15	Firmicutes	Clostridia	Clostridiales	Ruminococcaceae	Ruminococcus	flavefaciens	Ruminococcus flavefaciens
16	Firmicutes	Clostridia	Clostridiales				Clostridiales
17	Firmicutes	Clostridia	Clostridiales				Clostridiales
18	Bacteroidetes	Bacteroidia	Bacteroidales	S24-7			S24-7
19	Firmicutes	Clostridia	Clostridiales				Clostridiales
20	Firmicutes	Clostridia	Clostridiales	Lachnospiraceae			Lachnospiraceae
21	Bacteroidetes	Bacteroidia	Bacteroidales	S24-7			S24-7
22	Firmicutes	Clostridia	Clostridiales				Clostridiales
23	Firmicutes	Clostridia	Clostridiales				Clostridiales
24	Firmicutes	Clostridia	Clostridiales	Lachnospiraceae	Ruminococcus	gnavus	Ruminococcus gnavus
25	Firmicutes	Clostridia	Clostridiales				Clostridiales
26	Firmicutes	Clostridia	Clostridiales	Ruminococcaceae	Ruminococcus	flavefaciens	Ruminococcus flavefaciens
27	Firmicutes	Clostridia	Clostridiales				Clostridiales
28	Bacteroidetes	Bacteroidia	Bacteroidales	S24-7			S24-7
29	Bacteroidetes	Bacteroidia	Bacteroidales	Bacteroidaceae	Bacteroides		Bacteroides
30	Firmicutes	Clostridia	Clostridiales	Lachnospiraceae	Coprococcus		Coprococcus

31	Bacteroidetes	Bacteroidia	Bacteroidales	S24-7			S24-7
32	Firmicutes	Clostridia	Clostridiales	Lachnospiraceae			Lachnospiraceae
33	Firmicutes	Clostridia	Clostridiales	Ruminococcaceae	Oscillospira		Oscillospira
34	Firmicutes	Clostridia	Clostridiales	Ruminococcaceae			Ruminococcaceae
35	Firmicutes	Clostridia	Clostridiales				Clostridiales
36	Firmicutes	Clostridia	Clostridiales				Clostridiales
37	Firmicutes	Clostridia	Clostridiales				Clostridiales
38	Bacteroidetes	Bacteroidia	Bacteroidales	S24-7			S24-7
39	Firmicutes	Clostridia	Clostridiales	Ruminococcaceae	Oscillospira		Oscillospira
40	Firmicutes	Clostridia	Clostridiales	Lachnospiraceae	Dorea		Dorea
41	Firmicutes	Clostridia	Clostridiales				Clostridiales
42	Firmicutes	Clostridia	Clostridiales				Clostridiales
43	Bacteroidetes	Bacteroidia	Bacteroidales	S24-7			S24-7
44	Firmicutes	Clostridia	Clostridiales				Clostridiales
45	Firmicutes	Clostridia	Clostridiales				Clostridiales
46	Bacteroidetes	Bacteroidia	Bacteroidales	Bacteroidaceae	Bacteroides		Bacteroides
47	Bacteroidetes	Bacteroidia	Bacteroidales	S24-7			S24-7
48	Firmicutes	Clostridia	Clostridiales				Clostridiales
49	Firmicutes	Clostridia	Clostridiales	Lachnospiraceae			Lachnospiraceae
50	Actinobacteria	Coriobacteriia	Coriobacteriales	Coriobacteriaceae			Coriobacteriaceae
51	Bacteroidetes	Bacteroidia	Bacteroidales	S24-7			S24-7
52	Bacteroidetes	Bacteroidia	Bacteroidales	S24-7			S24-7
53	Bacteroidetes	Bacteroidia	Bacteroidales	S24-7			S24-7
54	Bacteroidetes	Bacteroidia	Bacteroidales	S24-7			S24-7
55	Bacteroidetes	Bacteroidia	Bacteroidales	Bacteroidaceae	Bacteroides		Bacteroides
56	Firmicutes	Clostridia	Clostridiales				Clostridiales
57	Firmicutes	Clostridia	Clostridiales				Clostridiales
58	Bacteroidetes	Bacteroidia	Bacteroidales	S24-7			S24-7
59	Bacteroidetes	Bacteroidia	Bacteroidales	Prevotellaceae	Prevotella		Prevotella
60	Proteobacteria	Alphaproteobacteria	RF32				RF32
61	Bacteroidetes	Bacteroidia	Bacteroidales	Bacteroidaceae	Bacteroides	ovatus	Bacteroides ovatus

62	Verrucomicrobia	Verrucomicrobiae	Verrucomicrobiales	Verrucomicrobiaceae	Akkermansia	muciniphila	Akkermansia muciniphila
63	Firmicutes	Clostridia	Clostridiales				Clostridiales
64	Bacteroidetes	Bacteroidia	Bacteroidales	S24-7			S24-7
65	Firmicutes	Clostridia	Clostridiales	Lachnospiraceae			Lachnospiraceae
66	Firmicutes	Clostridia	Clostridiales	Lachnospiraceae			Lachnospiraceae
67	Firmicutes	Clostridia	Clostridiales				Clostridiales
68	Proteobacteria	Betaproteobacteria	Burkholderiales	Alcaligenaceae	Sutterella		Sutterella
69	Bacteroidetes	Bacteroidia	Bacteroidales	Bacteroidaceae	Bacteroides		Bacteroides
70	Firmicutes	Clostridia	Clostridiales	Clostridiaceae	Candidatus Arthromitus		Candidatus Arthromitus
71	Firmicutes	Clostridia	Clostridiales				Clostridiales
72	Firmicutes	Clostridia	Clostridiales	Ruminococcaceae	Oscillospira		Oscillospira
73	Bacteroidetes	Bacteroidia	Bacteroidales	S24-7			S24-7
74	Firmicutes	Clostridia	Clostridiales				Clostridiales
75	Bacteroidetes	Bacteroidia	Bacteroidales	S24-7			S24-7
76	Firmicutes	Clostridia	Clostridiales	Lachnospiraceae			Lachnospiraceae
77	Firmicutes	Clostridia	Clostridiales	Ruminococcaceae	Oscillospira		Oscillospira
78	Firmicutes	Clostridia	Clostridiales	Ruminococcaceae			Ruminococcaceae
79	Bacteroidetes	Bacteroidia	Bacteroidales	Porphyromonadaceae	Parabacteroides		Parabacteroides
80	Firmicutes	Clostridia	Clostridiales	Ruminococcaceae	Oscillospira		Oscillospira
81	Bacteroidetes	Bacteroidia	Bacteroidales	S24-7			S24-7
82	Firmicutes	Clostridia	Clostridiales	Ruminococcaceae	Ruminococcus		Ruminococcus
83	Firmicutes	Clostridia	Clostridiales				Clostridiales
84	Bacteroidetes	Bacteroidia	Bacteroidales	S24-7			S24-7
85	Firmicutes	Clostridia	Clostridiales				Clostridiales
86	Firmicutes	Clostridia	Clostridiales				Clostridiales
87	Bacteroidetes	Bacteroidia	Bacteroidales	S24-7			S24-7
88	Firmicutes	Clostridia	Clostridiales	Ruminococcaceae	Oscillospira		Oscillospira
89	Firmicutes	Clostridia	Clostridiales				Clostridiales
90	Firmicutes	Clostridia	Clostridiales				Clostridiales
91	Bacteroidetes	Bacteroidia	Bacteroidales	S24-7			S24-7
92	Bacteroidetes	Bacteroidia	Bacteroidales	S24-7			S24-7

93	Firmicutes	Clostridia	Clostridiales	Ruminococcaceae			Ruminococcaceae
94	Bacteroidetes	Bacteroidia	Bacteroidales	S24-7			S24-7
95	Bacteroidetes	Bacteroidia	Bacteroidales	S24-7			S24-7
96	Firmicutes	Clostridia	Clostridiales				Clostridiales
97	Firmicutes	Clostridia	Clostridiales				Clostridiales
98	Firmicutes	Clostridia	Clostridiales				Clostridiales
99	Firmicutes	Clostridia	Clostridiales	Lachnospiraceae	Ruminococcus	gnavus	Ruminococcus gnavus
100	Bacteroidetes	Bacteroidia	Bacteroidales	Porphyromonadaceae	Parabacteroides	distasonis	Parabacteroides distasonis
101	Firmicutes	Clostridia	Clostridiales	Clostridiaceae			Clostridiaceae
102	Firmicutes	Clostridia	Clostridiales	Ruminococcaceae	Ruminococcus		Ruminococcus
103	Firmicutes	Clostridia	Clostridiales	Lachnospiraceae			Lachnospiraceae
104	Firmicutes	Clostridia	Clostridiales				Clostridiales
105	Firmicutes	Clostridia	Clostridiales				Clostridiales
106	Tenericutes	Mollicutes	RF39				RF39
107	Firmicutes	Clostridia	Clostridiales	Lachnospiraceae			Lachnospiraceae
108	Firmicutes	Clostridia	Clostridiales				Clostridiales
109	Firmicutes	Clostridia	Clostridiales	Lachnospiraceae			Lachnospiraceae
110	Firmicutes	Clostridia	Clostridiales				Clostridiales
111	Bacteroidetes	Bacteroidia	Bacteroidales	Porphyromonadaceae	Parabacteroides	distasonis	Parabacteroides distasonis
112	Firmicutes	Clostridia	Clostridiales	Lachnospiraceae			Lachnospiraceae
113	Firmicutes	Clostridia	Clostridiales	Ruminococcaceae	Oscillospira		Oscillospira
114	Firmicutes	Clostridia	Clostridiales	Lachnospiraceae			Lachnospiraceae
115	Firmicutes	Clostridia	Clostridiales				Clostridiales
116	Firmicutes	Clostridia	Clostridiales				Clostridiales
117	Firmicutes	Clostridia	Clostridiales				Clostridiales
118	Bacteroidetes	Bacteroidia	Bacteroidales	S24-7			S24-7
119	Firmicutes	Clostridia	Clostridiales				Clostridiales
120	Firmicutes	Clostridia	Clostridiales				Clostridiales
121	Bacteroidetes	Bacteroidia	Bacteroidales	Rikenellaceae			Rikenellaceae
122	Firmicutes	Clostridia	Clostridiales	Ruminococcaceae	Oscillospira		Oscillospira
123	Firmicutes	Erysipelotrichi	Erysipelotrichales	Erysipelotrichaceae	Allobaculum		Allobaculum

124	Firmicutes	Clostridia	Clostridiales				Clostridiales
125	Firmicutes	Clostridia	Clostridiales				Clostridiales
126	Bacteroidetes	Bacteroidia	Bacteroidales	Bacteroidaceae	Bacteroides		Bacteroides
127	Bacteroidetes	Bacteroidia	Bacteroidales	S24-7			S24-7
128	Bacteroidetes	Bacteroidia	Bacteroidales	S24-7			S24-7
129	Firmicutes	Clostridia	Clostridiales	Ruminococcaceae			Ruminococcaceae
130	Bacteroidetes	Bacteroidia	Bacteroidales	S24-7			S24-7
131	Firmicutes	Clostridia	Clostridiales	Ruminococcaceae			Ruminococcaceae
132	Firmicutes	Clostridia	Clostridiales				Clostridiales
133	Firmicutes	Clostridia	Clostridiales	Lachnospiraceae	Ruminococcus	gnavus	Ruminococcus gnavus
134	Firmicutes	Clostridia	Clostridiales	Ruminococcaceae			Ruminococcaceae
135	Bacteroidetes	Bacteroidia	Bacteroidales	S24-7			S24-7
136	Firmicutes	Clostridia	Clostridiales	Lachnospiraceae			Lachnospiraceae
137	Bacteroidetes	Bacteroidia	Bacteroidales	S24-7			S24-7
138	Firmicutes	Clostridia	Clostridiales	Clostridiaceae			Clostridiaceae
139	Firmicutes	Clostridia	Clostridiales				Clostridiales
140	Firmicutes	Clostridia	Clostridiales	Lachnospiraceae	Ruminococcus	gnavus	Ruminococcus gnavus
141	Bacteroidetes	Bacteroidia	Bacteroidales	S24-7			S24-7
142	Firmicutes	Clostridia	Clostridiales	Lachnospiraceae			Lachnospiraceae
143	Bacteroidetes	Bacteroidia	Bacteroidales	S24-7			S24-7
144	Cyanobacteria	4C0d-2	YS2				YS2
145	Bacteroidetes	Bacteroidia	Bacteroidales	S24-7			S24-7
146	Bacteroidetes	Bacteroidia	Bacteroidales	S24-7			S24-7
147	Firmicutes	Clostridia	Clostridiales	Lachnospiraceae			Lachnospiraceae
148	Firmicutes	Clostridia	Clostridiales				Clostridiales
149	Bacteroidetes	Bacteroidia	Bacteroidales	S24-7			S24-7
150	Firmicutes	Clostridia	Clostridiales				Clostridiales
151	Firmicutes	Clostridia	Clostridiales				Clostridiales
152	Bacteroidetes	Bacteroidia	Bacteroidales	S24-7			S24-7
153	Proteobacteria	Deltaproteobacteria	Desulfovibrionales	Desulfovibrionaceae	Desulfovibrio		Desulfovibrio
154	Firmicutes	Bacilli	Turicibacterales	Turicibacteraceae	Turicibacter		Turicibacter

155	Bacteroidetes	Bacteroidia	Bacteroidales	S24-7			S24-7
156	Firmicutes	Clostridia	Clostridiales	Lachnospiraceae			Lachnospiraceae
157	Bacteroidetes	Bacteroidia	Bacteroidales	S24-7			S24-7
158	Bacteroidetes	Bacteroidia	Bacteroidales	S24-7			S24-7
159	Firmicutes	Clostridia	Clostridiales				Clostridiales
160	Bacteroidetes	Bacteroidia	Bacteroidales	S24-7			S24-7
161	Bacteroidetes	Bacteroidia	Bacteroidales	Rikenellaceae			Rikenellaceae
162	Firmicutes	Clostridia	Clostridiales				Clostridiales
163	Bacteroidetes	Bacteroidia	Bacteroidales	S24-7			S24-7
164	Firmicutes	Clostridia	Clostridiales	Ruminococcaceae			Ruminococcaceae
165	Bacteroidetes	Bacteroidia	Bacteroidales	S24-7			S24-7
166	Firmicutes	Clostridia	Clostridiales				Clostridiales
167	Firmicutes	Clostridia	Clostridiales				Clostridiales
168	Proteobacteria	Betaproteobacteria	Burkholderiales	Alcaligenaceae	Sutterella		Sutterella
169	Bacteroidetes	Bacteroidia	Bacteroidales	S24-7			S24-7
170	Bacteroidetes	Bacteroidia	Bacteroidales	S24-7			S24-7
171	Bacteroidetes	Bacteroidia	Bacteroidales	S24-7			S24-7
172	Bacteroidetes	Bacteroidia	Bacteroidales	S24-7			S24-7
173	Bacteroidetes	Bacteroidia	Bacteroidales				Bacteroidales
174	Bacteroidetes	Bacteroidia	Bacteroidales	S24-7			S24-7
175	Bacteroidetes	Bacteroidia	Bacteroidales	S24-7			S24-7
176	Bacteroidetes	Bacteroidia	Bacteroidales	Bacteroidaceae	Bacteroides	acidifaciens	Bacteroides acidifaciens
177	Bacteroidetes	Bacteroidia	Bacteroidales	S24-7			S24-7
178	Firmicutes	Clostridia	Clostridiales				Clostridiales
179	Bacteroidetes	Bacteroidia	Bacteroidales	Odoribacteraceae	Odoribacter		Odoribacter
180	Bacteroidetes	Bacteroidia	Bacteroidales	S24-7			S24-7
181	Bacteroidetes	Bacteroidia	Bacteroidales	S24-7			S24-7
182	Bacteroidetes	Bacteroidia	Bacteroidales	S24-7			S24-7
183	Bacteroidetes	Bacteroidia	Bacteroidales	S24-7			S24-7
184	Bacteroidetes	Bacteroidia	Bacteroidales	S24-7			S24-7
185	Bacteroidetes	Bacteroidia	Bacteroidales	S24-7			S24-7

186	Bacteroidetes	Bacteroidia	Bacteroidales	S24-7		S24-7
187	Bacteroidetes	Bacteroidia	Bacteroidales	S24-7		S24-7
188	Bacteroidetes	Bacteroidia	Bacteroidales	S24-7		S24-7
189	Bacteroidetes	Bacteroidia	Bacteroidales	S24-7		S24-7
190	Bacteroidetes	Bacteroidia	Bacteroidales	S24-7		S24-7
