# Supplementary material for: Interleukin 1α-Deficient Mice Have an Altered Gut Microbiota Leading to Protection from Dextran Sodium Sulfate-Induced Colitis
Source: mSystems. 2018 May 8;3(3):e00213-17. doi: 10.1128/mSystems.00213-17 (PMC5940968; doi:10.1128/mSystems.00213-17)
Supplement: TABLE S2 [file sys003182227st2.pdf]

row number	Phylum	class	order	family	*genus*	*species*	lowest identified taxonomy
1	Firmicutes	Clostridia	Clostridiales				Clostridiales
2	Bacteroidetes	Bacteroidia	Bacteroidales	Rikenellaceae			Rikenellaceae
3	Firmicutes	Clostridia	Clostridiales	Lachnospiraceae			Lachnospiraceae
4	Firmicutes	Clostridia	Clostridiales				Clostridiales
5	Bacteroidetes	Bacteroidia	Bacteroidales	Bacteroidaceae	*Bacteroides*		*Bacteroides*
6	Bacteroidetes	Bacteroidia	Bacteroidales	S24-7			S24-7
7	Firmicutes	Clostridia	Clostridiales				Clostridiales
8	Firmicutes	Bacilli	Lactobacillales	Enterococcaceae	Enterococcus		Enterococcus
9	Firmicutes	Clostridia	Clostridiales	Ruminococcaceae	*Ruminococcus*	*gnavus*	*Ruminococcus gnavus*
10	Bacteroidetes	Bacteroidia	Bacteroidales	Bacteroidaceae	*Bacteroides*		*Bacteroides*
11	Firmicutes	Clostridia	Clostridiales				Clostridiales
12	Bacteroidetes	Bacteroidia	Bacteroidales	S24-7			S24-7
13	Proteobacteria	Gammaprote	Enterobacteriale	Enterobacteriaceae			Enterobacteriaceae
14	Bacteroidetes	Bacteroidia	Bacteroidales	Bacteroidaceae	*Bacteroides*		*Bacteroides*
15	Verrucomicrobia	Verrucomicr	Verrucomicrobia	Verrucomicrobiaceae	*Akkermansia*	*muciniphila*	*Akkermancia muciniphila*
16	Bacteroidetes	Bacteroidia	Bacteroidales	S24-7			S24-7
17	Bacteroidetes	Bacteroidia	Bacteroidales	S24-7			S24-7
18	Proteobacteria	Gammaprote	Enterobacteriale	Enterobacteriaceae			Enterobacteriaceae
19	Bacteroidetes	Bacteroidia	Bacteroidales	S24-7			S24-7
20	Firmicutes	Clostridia	Clostridiales	Lachnospiraceae			Lachnospiraceae
21	Bacteroidetes	Bacteroidia	Bacteroidales	Rikenellaceae			Rikenellaceae
22	Bacteroidetes	Bacteroidia	Bacteroidales	Bacteroidaceae	*Bacteroides*		*Bacteroides*
23	Bacteroidetes	Bacteroidia	Bacteroidales	S24-7			S24-7
24	Bacteroidetes	Bacteroidia	Bacteroidales	Bacteroidaceae	*Bacteroides*		*Bacteroides*
25	Firmicutes	Clostridia	Clostridiales	Ruminococcaceae			Ruminococcaceae
26	Firmicutes	Clostridia	Clostridiales				Clostridiales
27	Bacteroidetes	Bacteroidia	Bacteroidales	S24-7			S24-7
28	Firmicutes	Clostridia	Clostridiales	Ruminococcaceae			Ruminococcaceae
29	Firmicutes	Clostridia	Clostridiales				Clostridiales
30	Bacteroidetes	Bacteroidia	Bacteroidales	Bacteroidaceae	*Bacteroides*		*Bacteroides*

31	Firmicutes	Clostridia	Clostridiales			Clostridiales
32	Bacteroidetes	Bacteroidia	Bacteroidales	Bacteroidaceae	*Bacteroides*	*Bacteroides*
33	Firmicutes	Clostridia	Clostridiales			Clostridiales
34	Firmicutes	Clostridia	Clostridiales			Clostridiales
35	Firmicutes	Clostridia	Clostridiales			Clostridiales
36	Actinobacteria	Coriobacterii	Coriobacteriales	Coriobacteriaceae		Coriobacteriaceae
37	Bacteroidetes	Bacteroidia	Bacteroidales	Bacteroidaceae	*Bacteroides*	*Bacteroides*
38	Bacteroidetes	Bacteroidia	Bacteroidales	Bacteroidaceae	*Bacteroides*	*Bacteroides*
