# Supplementary figures and images for: Interleukin 1α-Deficient Mice Have an Altered Gut Microbiota Leading to Protection from Dextran Sodium Sulfate-Induced Colitis
Source: mSystems. 2018 May 8;3(3):e00213-17. doi: 10.1128/mSystems.00213-17 (PMC5940968; doi:10.1128/mSystems.00213-17)

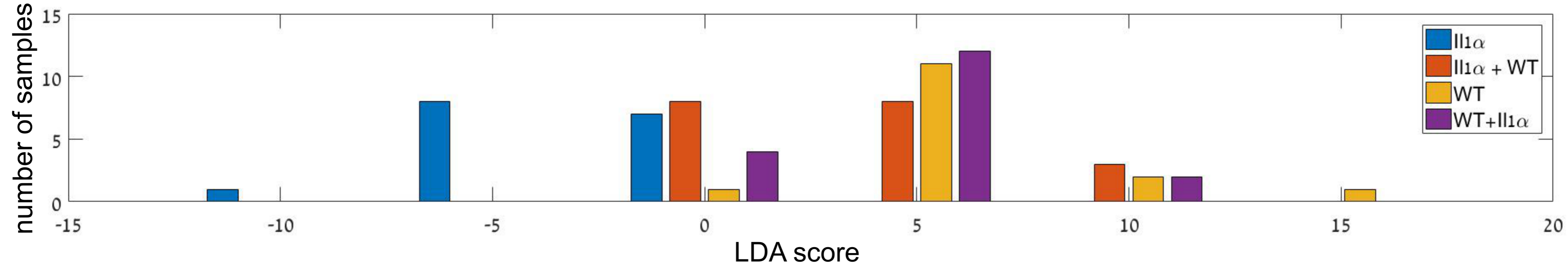

Supplement: FIG S1 [file sys003182227sf1.pdf]

A

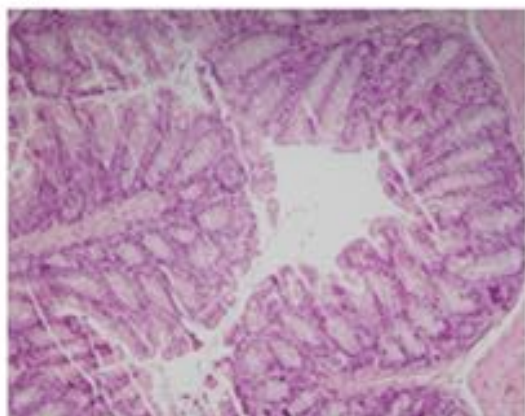

WT

B

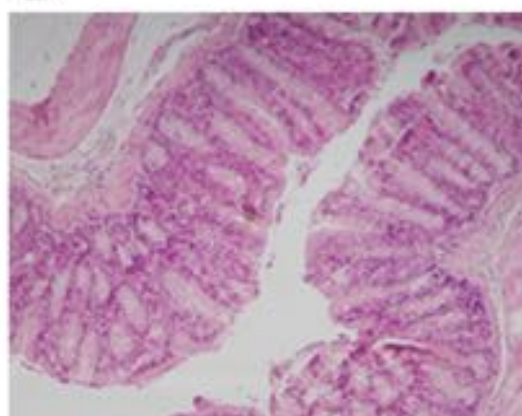

IL-1 $\alpha$  KO

Supplement: FIG S2 [file sys003182227sf2.pdf]
