# Supplementary material for: Interleukin 1α-Deficient Mice Have an Altered Gut Microbiota Leading to Protection from Dextran Sodium Sulfate-Induced Colitis
Source: mSystems. 2018 May 8;3(3):e00213-17. doi: 10.1128/mSystems.00213-17 (PMC5940968; doi:10.1128/mSystems.00213-17)
Supplement: TABLE S3 [file sys003182227st3.pdf]

Hotelling Results

	Cohousing_Day_0	Day_0	Day_i	Day_i_Cohousing	Day_8	Day_8_Cohousing	Day_14	Day_14_Cohousing
statistic	6E+00	1E+01	3E+01	6E+01	6E+00	3E+00	8E-01	1E-01
m	5E-01	5E-01	5E-01	5E-01	5E-01	5E-01	5E-01	5E-01
df	2, 35	2, 28	2, 29	2, 35	2, 20	2, 23	2, 14	1E+01
nx	2E+01	2E+01	2E+01	2E+01	2E+01	1E+01	9E+00	7E+00
ny	2E+01	2E+01	2E+01	2E+01	8E+00	1E+01	8E+00	9E+00
p	2E+00	2E+00	2E+00	2E+00	2E+00	2E+00	2E+00	2E+00
P_Value	6E-02	7E-03	1E-05	5E-08	9E-02	3E-01	7E-01	9E-01
Test stat	3E+00	6E+00	2E+01	3E+01	3E+00	1E+00	4E-01	6E-02

Benjamini Hochberg

5E-08	TRUE
1E-05	TRUE
7E-03	TRUE
6E-02	FALSE
9E-02	FALSE
3E-01	FALSE
7E-01	FALSE
9E-01	FALSE
